# An integrated model using the Taguchi method and artificial neural network to improve artificial kidney solidification parameters

**DOI:** 10.1186/s12938-019-0696-4

**Published:** 2019-07-05

**Authors:** An-Jin Shie, Kuei-Hsing Lo, Wen-Tsann Lin, Chi-Wen Juan, Yung-Tsan Jou

**Affiliations:** 1 School of Economics and Management, Huaiyin Normal University, No. 111, Changjiang West Road, Huaian, Jiangsu 223300 China; 2 Department of Industrial and Systems Engineering, Chung Yuan Christian University, 200 Chung Pei Road, Chung Li District, Taoyuan City, 32023 Taiwan; 3 Department of Industrial Engineering and Management, National Chin-Yi University of Technology, No. 57, Sec. 2, Zhongshan Road, Taiping District, Taichung City, 41170 Taiwan; 4 Medical Affairs, Kuang Tien General Hospital, No.117, Shatian Road, Shalu District, Taichung City, 433 Taiwan

**Keywords:** Hemodialysis, Artificial kidney solidification, Taguchi method, Omega transformation, Artificial neural network, Back-propagation network analysis

## Abstract

**Background:**

Hemodialysis mainly relies on the “artificial kidney,” which plays a very important role in temporarily or permanently substituting for the kidney to carry out the exchange of waste and discharge of water. Nevertheless, a previous study on the artificial kidney has paid little attention to the optimization of factors and levels for reducing the solidification of the artificial kidney during the hemodialysis procedure. Thus, this study proposes an integrated model that uses the Taguchi method, omega formula, and back-propagation network to determine the optimal factors and levels for addressing this issue.

**Methods:**

First, we collected the recommendations of medical doctors and nursing staff through a small group discussion, and used the Taguchi method to analyze the key factors at different levels. Next, the omega formula was used to convert the analysis results from the Taguchi method to assess the defect rate. Finally, we utilized back-propagation network algorithms to predict the optimal factors and levels for artificial kidney solidification, in order to confirm that the key factors and levels identified can effectively improve the solidification rate of the artificial kidney and thereby enhance the effect of hemodialysis.

**Results:**

The research finding proposes the following as the optimal factors and levels for artificial kidney solidification: the amount of anticoagulation should be set at 500 units, the velocity of blood flow at 300 ml/min, the dehydration volume at 2.5 kg, and the vascular access type as autologous blood vessels. We obtained 270 sets of data from the patients of End Stage Renal Disease (ESRD) under the setting of the optimal combination of the factors at different levels; the defect rate of artificial kidney solidification is 12.9%, which is better than the defect rate of 32% in the original experiment. Meanwhile, the patient characteristics for physiological status in BMI, serum calcium, hematocrit, ferritin, and transferrin saturation percentage are improved by this study.

**Conclusion:**

This conclusion validates the ability of the proposed model in this study to improve the solidification rate of the artificial kidney, thereby confirming the model’s use as a standard operation procedure in the hemodialysis experiment. The ideas behind and the implications of the proposed model are further discussed in this study.

## Background

Kidney disease is a type of chronic disease. According to the Department of Health, the Executive Yuan, kidney disease was the fifth leading cause of death in Taiwan in 2014. Once the renal function has progressed to the uremic stage, the patient needs to rely on an artificial kidney to carry out hemodialysis treatment for the rest of his/her life or undergo a kidney transplantation in order to survive [1]. Currently, there are more than 230,000 hemodialysis patients in Taiwan (in 2014 statistics). The hemodialysis treatment is carried out by an artificial kidney, the functions of which include the discharge of dissolved uremic toxins and water. Thus, the dialyzer plays a very important role in the process of hemodialysis [2].

In the past few years, the medical industry has become increasingly competitive. With the rise in consumer consciousness, it has become even more imperative for all hospitals to enhance the quality of medical services and patient satisfaction. In terms of the current medical sector, quality management and other methods are most commonly used to solve the issues related to medical service quality and process. For example, Shen et al. [3] and Lin et al. [4] discussed the use of a quality control circle to approach issues related to the effective enhancement of the workflow efficiency and cost reduction of clinical hemodialysis. Matías-Guiu et al. [5] used the Kano two-dimensional model to determine the important factors of seeking medical treatment, such as the quality perception of and patient satisfaction from the hemodialysis medical service. In addition, Rezapour et al. [6] and Kusiak et al. [7] applied the data mining method to the treatment records of hemodialysis patients to forecast the patients’ survival rates, with the aim of providing the optimal medication plan and lowering the patients’ expenses.

Although these studies and their proposed methods all help to enhance the quality of medical services, there remains a lack of research on the factors that affect the solidification rate of an artificial kidney. As a result, professional doctors and nursing staff might not know how to apply scientific methods to choose key factors when they implement hemodialysis in an experiment. In practice, in most cases, the experimentation of the parameters for artificial kidney solidification relies on the subjective experiences of individual doctors and nurses, which results in substantial differences in the solidification rate of an artificial kidney, and hence the reduction of patient survival rates [8]. They needed an establishment of optimal factors and levels by a scientific method for reducing the artificial kidney solidification rate.

Thus, in order to solve the questions relating to the solidification rate of an artificial kidney, this study proposes the use of a model that integrates the Taguchi method, omega transformation, and back-propagation network to determine the key factors and levels for conducting hemodialysis with an artificial kidney.

### Purposes of the study

This study conducted a case study on the setting parameters currently used by a hospital’s dialysis unit when implementing hemodialysis with an artificial kidney. Data on artificial kidney solidification collected by a certain hospital’s dialysis unit were analyzed, and through a small group discussion method, the experiences of the professional nursing staff and doctors in the dialysis unit were systematically collected, in order to facilitate the formulation of the important factors of artificial kidney solidification.

The study process mainly consists of the application of the Taguchi method and omega transformation to analyze the optimal parameter design for artificial kidney solidification; a confirmation experiment to confirm whether the artificial kidney solidification rate is decreased; and finally, the back-propagation network verification to verify the convergence of the results. The analysis results can be used as a reference for the establishment and development of competition strategies by players in the medical industry. The purposes of this study are as follows:

- Find out the optimal factors and levels affecting the artificial kidney solidification rate during the hemodialysis experiment by proposing an integrated model.
- Provide combinations of key factors and levels of the operational procedure by reviewing and analyzing previous literature and doctors’ professional experiences as well as applying the Taguchi experimental method and back-propagation network to decrease artificial kidney solidification, which has implications for medical institutions when conducting hemodialysis.
- Develop an optimal experiment process based on the Taguchi method and obtain the optimal model for further analysis through the omega transformation to prevent the artificial kidney from solidifying and to reduce the consumption of the artificial kidney.

## Literature review

Based on the aforementioned research background and purposes, this study mainly seeks to propose an integrated model using the Taguchi method and to discuss further the issues related to the decrease of the artificial kidney solidification rate. Thus, in this section, we will introduce the issues related to hemodialysis and its implementation procedures, and artificial kidney solidification.

### Hemodialysis

Hemodialysis is the American approach of treating end-stage renal disease. In the past decade, patients who voluntarily chose the hemodialysis treatment were mostly older patients, diabetic patients, patients with more concurrent symptoms, and patients with artery stiffening cardiovascular disease [9].

Long-term dialysis can be divided into hemodialysis and peritoneal dialysis. Concerning the distribution of these two types of dialysis methods, hemodialysis is currently the main treatment method: over 90% of end-stage renal disease patients use hemodialysis, while the remaining 10% of patients use peritoneal dialysis [9, 10]. According to the Annual Report by Taiwan Society of Nephrology, up until the end of 2005, 90% of patients received hemodialysis treatment in Taiwan [11]. Hemodialysis is the treatment process that uses patients’ blood for extracorporeal circulation, thereby improving electrolyte and abnormal pH imbalance and helping patients with uremic symptoms. Hemodialysis is mainly used to treat chronic and acute renal function failure management, which is generally difficult to treat with traditional medicine [12].

#### Hemodialysis procedures

**Fig. 1:**
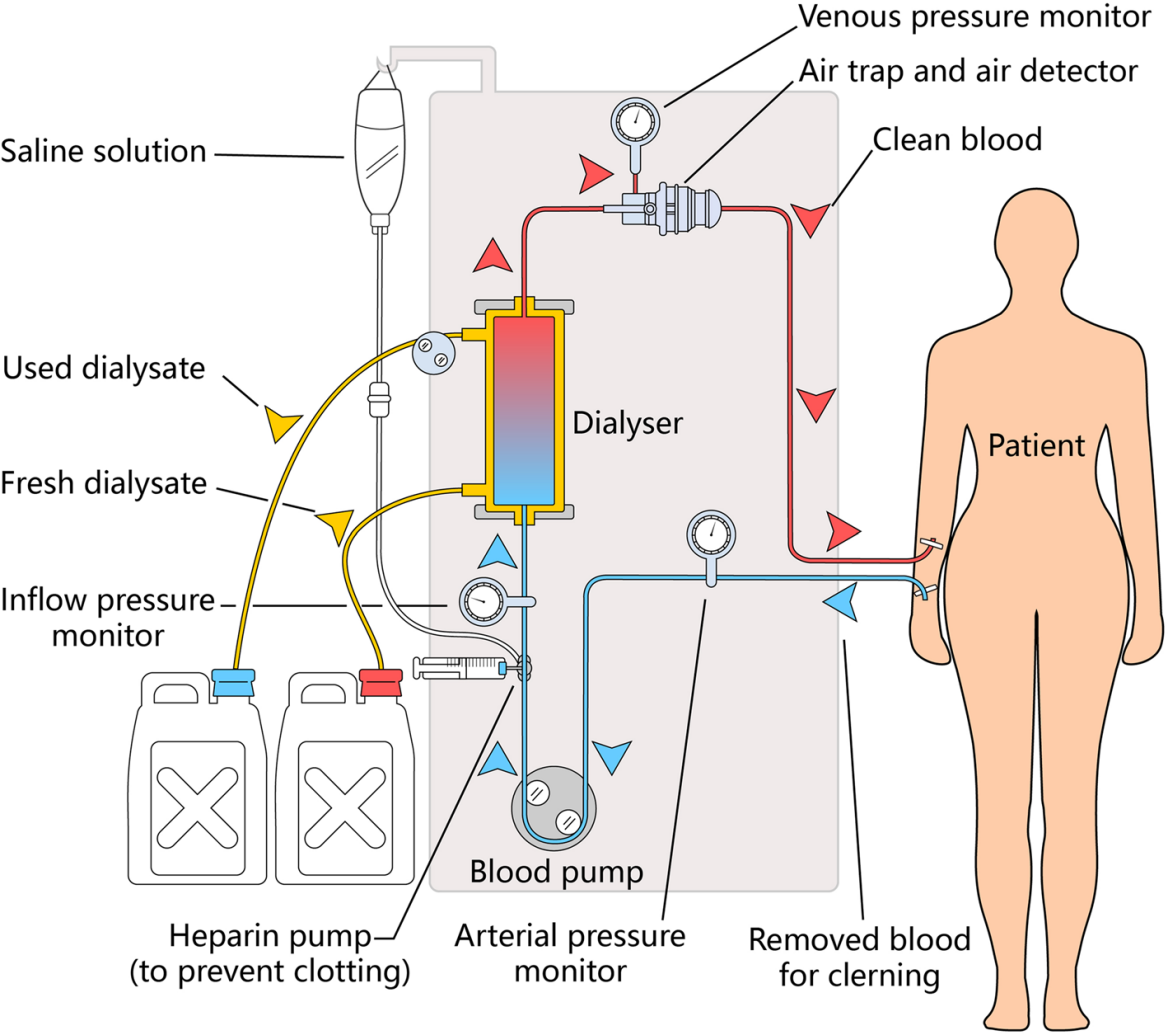
Hemodialysis procedures

When performing hemodialysis, the nursing staff inserts two needles into the patient (see Fig. 1). The first needle is used to drain blood out of the patient. The hemodialysis machine is then used to bring the blood to the artificial kidney via the artery vessel passage, and the blood is then cleansed through the artificial kidney. There are tens of thousands of hollow fiber tubes, the material of which consists of a semi-permeable membrane with tiny holes. These substitute for the patient’s kidney through the principle of diffusion. The blood will first fill up each fiber tube within the artificial kidney and then move from one end to the other end. Next, the clean dialysis solution will be injected into the other end of the artificial kidney and then fill up the peripheral of all fiber tubes. The blood and the dialysis solution thus exchange materials through the semi-permeable membrane, taking advantage of the principle of osmosis. The metabolic wastes in the blood will diffuse to the clean dialysis solution, thereby achieving the purpose of blood purification [13]. The other needle is used to allow the purified blood to flow back to the patient’s body through the artery vessel passage. Repeating the above cycle and the overall hemodialysis treatment takes about 4–5 h to complete [14].

#### Factors affecting the quality of hemodialysis

There are many important factors that affect the medical effect of hemodialysis. After a review and analysis of the previous literature, this study has discovered that the factors can be divided into four dimensions, such as patient demographic factors, patient health and physiological factors, and medical care factors [15, 16]. The details are as follows:

- Patient demographic factors: patient’s gender, patient’s age, and residence.
- Patient health factors: primary disease, status of blood pressure, blurred vision/blindness, physical disorder, unconsciousness, discomfort or lack of discomfort during the dialysis process, and previous medical history.
- Patient physiological factors: Body Mass Index (BMI), serum calcium, hematocrit, ferritin, and transferrin saturation percentage.
- Medical care factors: the amount of anticoagulation, the vascular access type, the size of the membrane in the artificial kidney, the material characteristics of the fiber in the artificial kidney, the dehydration ratio of the artificial kidney, the size of the blood flow provided by the “dialysis duct” or “arteriovenous fistula,” the velocity of flow of the dialysis solution, the time needed for hemodialysis, the hourly dehydration rate set by the dialysis machine, the length of time of using the patient’s artery vessel, and the temperature set by the dialysis machine.

### Solidification of the artificial kidney

#### Factors affecting artificial kidney solidification

After the blood is in contact with air, the protein will adhere to the pipeline, after which the platelets will accumulate continually. This is one of the main causes of the solidification of the artificial kidney during the hemodialysis procedure. Clinically, common causes of the solidification of the artificial kidney include insufficient blood flow in the arteriovenous fistula, an excessively high hematocrit value, and the presence of air in the artificial kidney or blood transfusion through the duct loop [13, 14].

#### Impacts of the solidification of the artificial kidney

Clinically, the artificial kidney solidification rate is about 1%. When the anticoagulation is insufficient or the technical operation is improper, the artificial kidney solidification ratio can be as high as 14% or above. During the hemodialysis treatment, both the artificial kidney and the pipelines should be observed at all times to see if there is any blood coagulation, especially in patients undergoing hemodialysis procedures without any anticoagulation [17, 18]. In addition to the waste of material costs (NTD 1885/unit) and the disposal costs of the derived medical waste, artificial kidney solidification interrupts the hemodialysis and affects the dialysis purification rate. When the blood remains in the pipeline or the artificial kidney, it can cause a loss of blood of up to 200 CC–240 CC in patients as well as clinical symptoms such as dizziness, drowsiness, palpitation during activities, and severe anemia. These effects can lead to concern and complaints from the patients and their family members, which in turn creates distrust toward medical care and questions about the quality and safety of dialysis procedures. Therefore, an effective reduction of the artificial kidney solidification rate leads to not only the reduction of related material costs and the disposal costs of the derived dialysis waste but also the enhancement of the survival rate of patients undergoing hemodialysis procedures [8, 14].

## The proposed model and research method

**Fig. 2:**
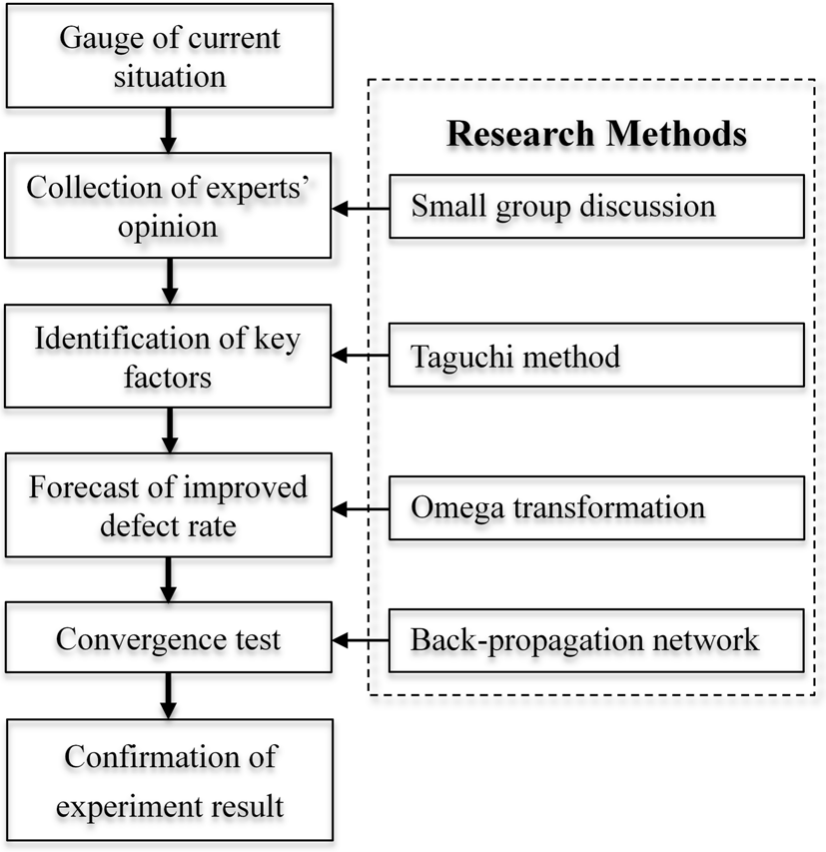
The proposed integrated model

This section focuses on the research model proposed by this study and its relevant theoretical methods: the Taguchi method, omega transformation, and back-propagation network. First, this study will analyze the optimal factors and levels for artificial kidney solidification and propose an integrated model, as shown in Fig. 2.

We used a small group discussion method to systematically gather professional recommendations from doctors and nursing staff. We then extracted the important factors affecting artificial kidney solidification and used the Taguchi method to design the artificial kidney solidification experiment in order to identify the optimal combination of factors and levels. Next, the omega formulation was used to conduct the omega transformation of the Taguchi analysis results, leading to a confirmation of the defect rate. Finally, this study used the back-propagation network method to assess the analysis results from the Taguchi experiment. The operational steps of the proposed model are as follows:

1. Small group discussion: extract the important key factors and levels of artificial kidney solidification from professional doctors and nursing staff via a group discussion.
2. Taguchi method: identify the quality characteristics of the artificial kidneys, establish the Taguchi orthogonal array experiment, and analyze the factor and level effects to obtain the optimal combination of factors and levels.
3. Omega transformation: transform the binary data (good quality or defective quality) generated by the proposed optimal combination of factors and levels into dB (the unit of the Signal-to-Noise ratio) to estimate the defect rate.
4. Artificial neural network: utilize the back-propagation network to predict whether the proposed combination of optimal factors and levels is stable with low margins of error.
5. Confirm the analysis: conduct an actual hemodialysis procedure to confirm whether the proposed optimal factors and levels have good-quality reproducibility in the hemodialysis experiment.

### The Taguchi method

The Taguchi Method is a quality-control engineering design proposed by Dr. Genichi Taguchi in the 1950s and early 1960s. It is also known as the Taguchi quality engineering or Taguchi experimental design method. As it is quite similar to the construction method, it is also called a robust design. Its main purpose is to provide innovative and efficient construction technology in terms of quality design to improve productivity. The Taguchi method determines design parameters by means of “experiments,” the definition of which is broad here, as it can be experiments in a laboratory, experiments within a factory production line, or a computer simulation experiment [19]. The Taguchi method offers a set of the scientific experimental procedures which help the researcher to systematically establish optimizing experimental factors and levels and to achieve a reliable estimated value of factors in fewer experiments. It can improve the solidification of the artificial kidney during the hemodialysis procedure.

#### Parameter design

The parameter design takes advantage of experiments to determine the combination of the controlling factor and level; it is one of the many quality control methods proposed by Taguchi, and it has achieved the greatest contribution in terms of enhancing quality [20, 21]. The design takes advantage of the principle of the orthogonal array, using fewer experiments and a simple configuration of the experiment by reducing the sensitivity of the system to the noise factor. This enhances the robustness of the system and demonstrates the reproducibility of the experiment result. Hence, once the quality of the product or the production process is improved, a similar result will continue to appear in future productions. This method was later widely used in various other industries. With the parameter design, we can identify a set of the optimal parameter combination, so that the average value of the quality characteristics is consistent with the target value, and the variation is kept to a minimum. The parameter design is a kind of technology improvement rather than a type of scientific research, and is currently one of the best methods to improve product quality in the industry.

In the parameter design, the nonlinear and linear relationships between the controlling factors and the noise factors are used. First, the variation is reduced by taking advantage of the nonlinear relationship. Next, the average value of the characteristics is adjusted to the target value by taking advantage of the linear relationship. By changing the standard of the factors, the variation of the quality characteristics is significantly reduced, thereby reducing the improvement costs and enhancing the quality of the product.

#### Orthogonal array

**Fig. 3:**
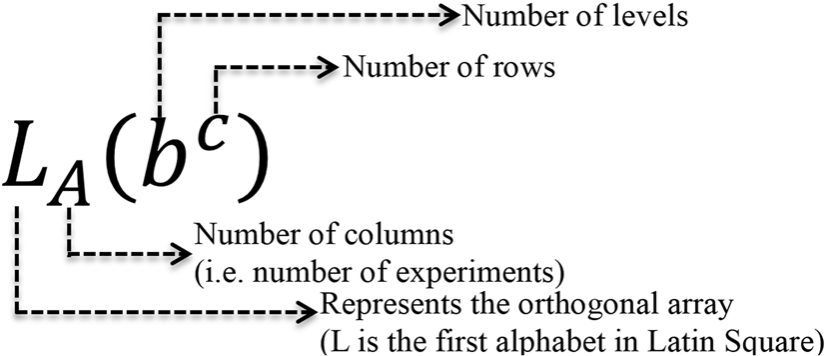
Basic introduction of the orthogonal array

In the full factorial design, when the number of factors increases, the number of experiments increases rapidly. Through the experiments arranged by the orthogonal array, we can acquire a reliable estimated value of factors with fewer experiments. Thus, the orthogonal array is an important technique to use to conduct experiments of a robust design. In consideration of the stability and cost factor of products, the orthogonal array is an important tool for engineers when they conduct the assessment of product and production process designs. The orthogonal array symbols and the meanings they represent are shown in Fig. 3 [20, 21].

### Neural network

The neural network is a science that uses computers to simulate the neural structure of animals and the neural cell network of humans by creating parallel computing patterns. Accumulative experience is acquired from past environmental messages and converted into knowledge to be stored. An intelligent computing procedure is then established to take advantage of the stored knowledge. It is an important branch of artificial intelligence and can be used for follow-up prediction or identification purposes. People are gradually beginning to understand the thinking patterns and learning patterns of the brain, so questions are solved by taking advantage of the computer’s computing power [22].

#### Back-propagation network

The input layers of the neurons are used to import external messages, the output layers of the neurons are used to export internal messages, and the hidden layers are used to process the interactions among neurons [23].

More specifically, input layers are used to represent the input variables of the network, and the number of processing units depends on the characteristics of a given question. The input variables of this study were established as the important factors derived from the Taguchi method analysis, including the amount of anticoagulation, the velocity of blood flow, the dehydration volume, and the vascular access type. These four parameters were used as the input variables of the back-propagation network.

Hidden layers represent the interactions among the input processing units, and there are no standard rules to determine the number of processing units. The optimal number is usually determined by the experimental method, and the nonlinear functions are used. The network could lack hidden layers or include more than one hidden layer.

Output layers present the output variables of the network, and the number of processing units also depends on the question. The nonlinear conversion functions are still used. The output variable in this experiment was established as the data of artificial kidney solidification.

#### Hidden-layer parameter setting

When the back-propagation network method is used, the optimal number is mostly determined through the experimental method, and the back-propagation network allows the hidden layer to be set as zero or multiple. This study mainly calculated the setting of the number of hidden layers through the conversion functions. When good convergence is desired, there must be one to two hidden layers. Based on the discussion of a previous study, general questions only need one hidden layer for the research to be conducted. The number of neurons can determine the level of questions, such as simple questions, general questions, or difficult questions.

- Simple questions = average method: (the number of input-layer processing units + the number of output-layer processing units)/2 = 3.
- General questions = summation method: (the number of input-layer processing units + the number of output-layer processing units) = 5.
- Difficult questions: doubling method: (the number of input-layer processing units + the number of output-layer processing units) × 2 = 10.

In this experiment, as there were four input units and one output-layer processing unit; the sum was 5, rendering it a general question. Based on the discussion of a previous study, the hidden layer was set as one layer. In addition, this study used the back-propagation network to predict the combination of optimal factors and levels for achieving stable results due to the network’s following advantages: (i) compared with the traditional statistical modeling method, which is limited by many assumptions, its range of application is wide; (ii) it can handle complicated sample identification problems; (iii) it can handle highly nonlinear functions; (iv) its response speed is fast; and (v) it allows different kinds of variables to be used as input variables [22].

## Case study

### Selecting the quality characteristics

**Fig. 4:**
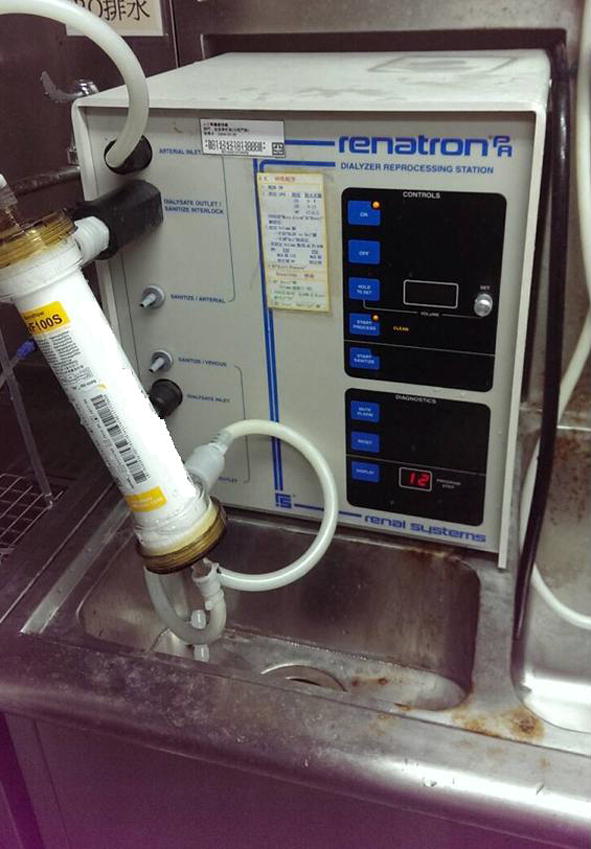
Artificial kidney capacity detection

In this study with repeatedly used artificial kidneys as the research object, the proposed integrated model was used to improve the coagulation ratio of the artificial kidneys. We adopted the count value as the quality characteristics. The artificial kidney capacity detection equipment was utilized to identify whether the usable volume in the artificial kidney was too low (see Fig. 4), and distinguish a good or defective quality of the artificial kidneys coagulation.

**fig. 5:**
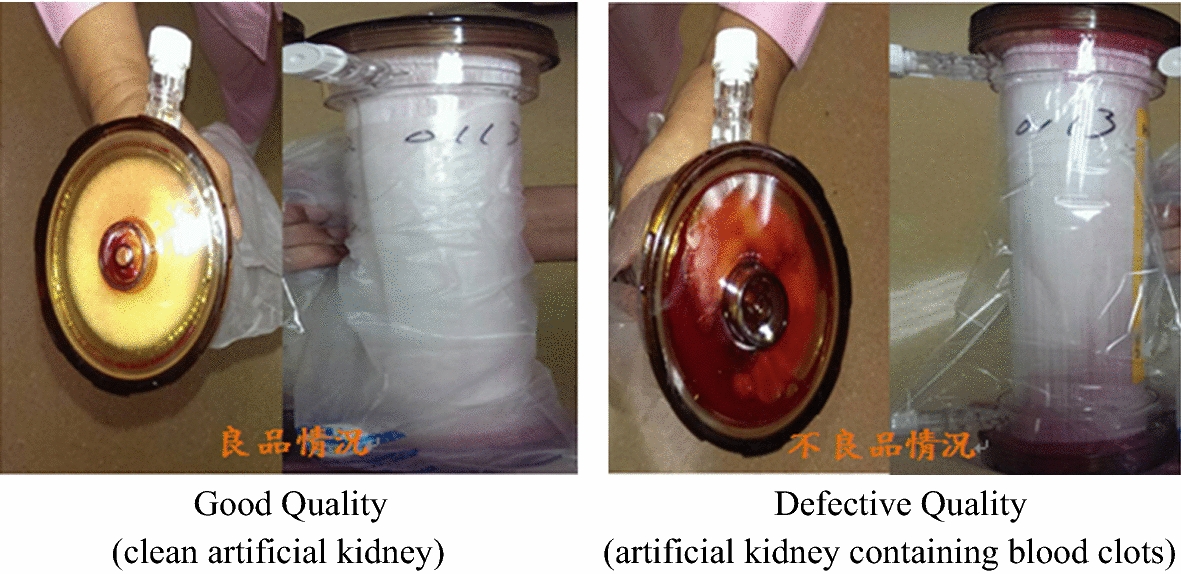
Good and defective artificial kidneys

The undesirable condition of the solidification of artificial kidneys was defined as follows: when 20% of the artificial kidney contains blood clots, the artificial kidney is regarded as having a defective quality, which means that the repeated-use fill volume is insufficient. In contrast, when blood clots take up less than 20% of the artificial kidney, the artificial kidney is regarded as having a good quality. Depending on the degree of impact on their quality, the artificial kidneys are divided into having a good or defective quality [1, 15], as shown in Fig. 5.

In this study, we used the international standards of the repeated-use fill volumes of artificial kidneys, as shown in Table 1. The standards list six types and repeated-use fill volumes, the most commonly used of which include F-100, F-80, FX-100, FX-80, FX-80M, and FX-60M. These are regarded as references for confirming good and defective qualities in repeatedly used artificial kidneys.

**Table 1:** International standards of the repeated-use fill volumes of artificial kidneys

Type of artificial kidney	F-100	F-80	FX-100	FX-80	FX-80M	FX-60M
100% volume (original)	132	110	106	88	95	74
80% volume (repeated use)	106	88	85	70	76	59

Volume units are in CC

For example, we used the “artificial kidney capacity” detection equipment to confirm whether the usable volume in the F-100 artificial kidney was too low. According to the artificial kidney solidification rate detection, if 80% of the remaining content volume is usable (or the volume is greater than 106 cc), if the volume achieves or exceeds the standards shown in Table 1, then it is qualified. To enhance the rigorousness of the research results, this study conducted the artificial kidney solidification volume experiment entirely in accordance with the international standards of the repeated-use fill volumes of artificial kidneys.

### The selection of factors and levels

The small group discussion method and cause-and-effect charts were conventionally used to conduct the selection of key factors, and then the Taguchi method was applied to identify key factors in order to avoid the negligence and blind points within the investigators’ personal consciousness. Nevertheless, there are too many factors and parameter levels involved in the coagulation of a hemodialyzer; processing them via the Taguchi methods would consume substantial time and costs. Some of the previous literature have not controlled for the factors of coagulation. Further, some are not fit for alteration, which might produce biased experimental outcomes. Thus, this study used Konduk and Ucisik [24] and Singh et al. [25] suggestions to integrate the opinions of the professionals to exclude the aforementioned factors and applied the Taguchi experimental designs to the remaining factors. In the end, the authors discussed with the professional doctors and nursing staff to obtain four factors and three levels as shown in Table 2.

**Table 2:** Factors at different levels

Name of factors	Parameter levels		
	1	2	3
Amount of anticoagulation (units)	500	1000	1500
Velocity of blood flow (ml/min)	200	250	300
Dehydration volume (kg)	1.5	2.5	3.5
Vascular access type	Artificial blood vessel	Autologous blood vessel	Temporary duct

Dehydration is usually used in intervals in clinical practice

### Orthogonal array experiment configuration and analysis

In this study, we also utilized three experiment levels for each of the four factors selected. As the total degree of freedom (*df*) of this experiment is 8, we chose the L9 (3^4^) orthogonal array to conduct the experiment by arranging factors A, B, C, and D with rows 1, 2, 3, and 4, as shown in Table 3.

**Table 3:** The L9 (3^4^) orthogonal array

A	B	C	D
1	1	1	1
1	2	2	2
1	3	3	3
2	1	2	3
2	2	3	1
2	3	1	2
3	1	3	2
3	2	1	3
3	3	2	1

### Data collection

In this study, we adopted the international standards of the repeated-use fill volumes of artificial kidneys (see Table 1). The quality of hemodialysis was divided into two categories: when 20% of the artificial kidney is solidified (or when the fill volume is less than 80%), the kidney is considered to be of a defective quality; when the solidification is less than 20% (or when the fill volume is 80% or more), the kidney is considered to be of a good quality. We applied L9 (3^4^) to the experiment. We conducted 30 trials consisting of each combination of the four factors at three levels in the hemodialysis procedure. Meanwhile, the experimental study was conducted in accordance with guidelines proposed by the Basic & Clinical Pharmacology & Toxicology (BCPT) policy for experimental and clinical studies [26]. The data collection process suggested by Fayed et al. [9] was applied in this study, the data collection aimed at the patient of the End Stage Renal Disease (ESRD) receiving the regular hemodialysis treatment (note: hemodialysis of two to three times within a week) for at least 3 months. The patients selected were diagnosed by the doctors as patients of end-stage renal disease, and they need intensive care service. Thus, we obtained 270 sets of data from the patients who have experienced the experiment. The defect rate is 32%, and the experimental data records are shown in Table 4.

**Table 4:** Experimental data

Experiment no.	A	B	C	D	Defective quality	Good quality	Detection sample
1	1	1	1	1	7	23	30
2	1	2	2	2	6	24	30
3	1	3	3	3	10	20	30
4	2	1	2	3	10	20	30
5	2	2	3	1	16	14	30
6	2	3	1	2	9	21	30
7	3	1	3	2	11	19	30
8	3	2	1	3	12	18	30
9	3	3	2	1	5	25	30
Total					86	184	270

The patient characteristics for health status are summarized in Table 5, regarding residence types, gender, age, primary disease, unstable blood pressure, blurred vision/blindness, physical disorder, and unconsciousness. Of the 270 sets, patient characteristics were obtained in the research sample: 35.1% lived downtown (*n* = 95), 55.6% lived rural area (*n* = 150), and 9.3% lived fishing village (*n* = 25). Approximately 46.9% of the patients (*n* = 127) were male, and 53.1% (*n* = 143) were female. In terms of the age of the patients, 17.9% (*n* = 48) were less than 50-year old, 33.2% (*n* = 90) were within the range of 51- to 64-year old, 20.6% (*n* = 56) were within the range of 65- to 74-year old, and 28.3% (*n* = 76) were 75-year old and above. In terms of the primary disease of the patient, 62.1% (*n* = 168) were chronic glomerulonephritis, and 36.9% (*n* = 100) were diabetes. In terms of unstable blood pressure of the patient, 41.10% (*n* = 111) had unstable blood pressure, and 58.90% (*n* = 159) had stable blood pressure. In terms of blurred vision/blindness of the patient, 28.30% (*n* = 76) had blurred vision/blindness, and 71.70% (*n* = 194) did not have blurred vision/blindness. In terms of physical disorder of the patient, 13.7% (*n* = 37) were physical disorder, and 86.3% (*n* = 233) were no physical disorder. Of the patient, 1.90% (*n* = 5) were unconsciousness, and 98.10% (*n* = 265) were clear consciousness.

**Table 5:** The patient characteristics’ (*n* = 270) analysis for health status

Characteristics	Variables	Sample size	Percentage
Residence types	Downtown	95	35.1
	Rural area	150	55.6
	Fishing village	25	9.3
Gender	Male	127	46.9
	Female	143	53.1
Age	Less than 50 years old	48	17.9
	51–64 years old	90	33.2
	65–74 years old	56	20.6
	75 years old and above	76	28.3
Primary disease	Chronic glomerulonephritis	168	62.1
	Diabetes	100	36.9
	Others	2	1.0
Unstable blood pressure	Yes	111	41.10
	No	159	58.90
Blurred vision/blindness	Yes	76	28.30
	No	194	71.70
Physical disorder	Yes	37	13.70
	No	233	86.30
Unconsciousness	Yes	5	1.90
	No	265	98.10

The patient characteristics for physiological status are summarized in Table 6, regarding Body Mass Index (BMI), serum calcium, hematocrit, ferritin, and transferrin saturation percentage. Of the 270 patients, 24.4% (*n* = 39) were low BMI less than 18 kg/m^2^, 34.5% (*n* = 174) were normal BMI within the range of 18–24 kg/m^2^, and 41.1% (*n* = 57) were high BMI greater than 24 kg/m^2^. In terms of serum calcium of the patient, 14.6% (*n* = 12) were low level less than 8.5 mg/dl, 49.2% (*n* = 187) were normal level within the range of 8.5–10.5 mg/dl, and 36.2% (*n* = 71) were high level greater than 10.5 mg/dl. In terms of the hematocrit of the patient, 6.5% (*n* = 18) were low hematocrit level less than 25%, 20.1% (*n* = 54) were normal hematocrit level within the range of 25–30%, and 73.4% (*n* = 198) were high hematocrit level greater than 30%. In terms of the ferritin of the patient, 23.9% (*n* = 65) were low ferritin level less than 300 mg/dl, 65.9% (*n* = 178) were normal ferritin level within the range of 300 to 800 mg/dl, and 10.2% (*n* = 27) were high ferritin level greater than 800 mg/dl. Of the patients, 27.8% (*n* = 48) were the low percentage of transferrin saturation less than 20%, and 72.2% (*n* = 222) were the normal percentage of transferrin saturation greater than 20%.

**Table 6:** The patient characteristics’ (*N* = 270) analysis for physiological status

Characteristics	Variables	Sample size	Percentage
Body Mass Index (BMI)	< 18 kg/m^2^ (low)	39	24.4
	18–24 kg/m^2^ (normal)	174	34.5
	> 24 kg/m^2^ (high)	57	41.1
Serum calcium	< 8.5 mg/dl (low)	12	14.6
	8.5–10.5 mg/dl (normal)	187	49.2
	> 10.5 mg/dl (high)	71	36.2
Hematocrit	< 25% (low)	18	6.5
	25–30% (normal)	54	20.1
	> 30% (high)	198	73.4
Ferritin	< 300 mg/dl (low)	65	23.9
	300–800 mg/dl (normal)	178	65.9
	> 800 mg/dl (high)	27	10.2
Transferrin saturation percentage	< 20% (low)	48	27.8
	> 20% (normal)	222	72.2

### The effect of each factor and its corresponding accumulated probability

Based on the orthogonal array table, for factor A at level 1, the sum of the detection value collected in the first, second, and third experiments in the accumulated category (defective quality) equals 7 + 6+10 = 23. By the same token, for standard A1, the same collected in the accumulated category (detection total) is 30 + 30 + 30 = 90. The same calculation can be applied to the remaining factors in this way. After this step is completed, we can continue to calculate the corresponding accumulated probability via the following computing method: for factor A at level 1 in the accumulated category (defective quality), the probability is 23/90 = 0.26; for factor A at level 1 in the accumulated category (detection total), the probability is 90/90 = 1.00. The remaining factors at different levels can be calculated in the same way, as shown in Table 7.

**Table 7:** Factor and level effects

Factor	Level	Detection value in accumulated category	Accumulated probability
		(Defective quality)	(Defect rate)
A	A1	23	0.26
	A2	35	0.39
	A3	28	0.31
B	B1	28	0.31
	B2	34	0.38
	B3	24	0.27
C	C1	28	0.31
	C2	21	0.23
	C3	37	0.41
D	D1	28	0.31
	D2	26	0.29
	D3	32	0.36

Detection total is 90

**Fig. 6:**
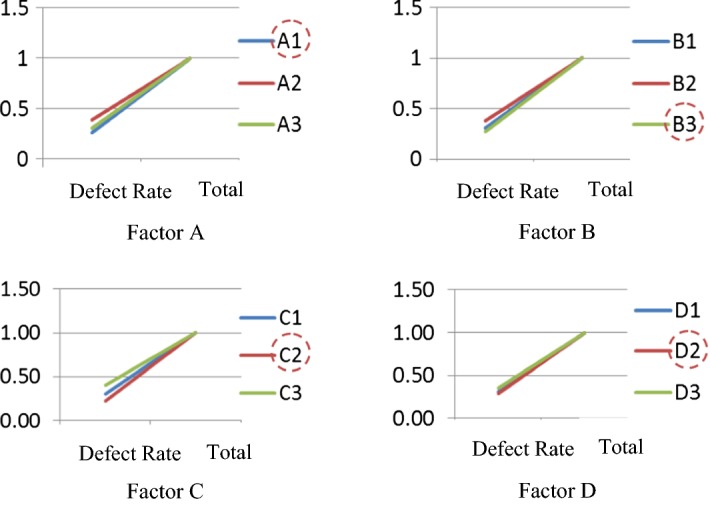
The effect diagram of the four factors

Using the effect diagram of the four factors (see Fig. 6), this study adopted “the smaller, the better” quality characteristics in the experimental design. For factor A, we know that the defect rate of A1 is lower than the defect rates of A2 and A3, so A1 is regarded as the main factor and level. For factor B, the defect rate of B3 is lower than the defect rates of B1 and B2, so B3 is the main factor and level. The same logic was applied to factors C and D to deduce the main factor and level. Finally, using the line chart of factor effects with the aim of reducing the artificial kidney solidification rate (the lower the defect rate, the better), we found that the optimal controlling factor and level combination is A_1_B_3_C_2_D_2_, which can be used as a benchmark condition for the artificial kidney solidification rate in the future. Thus, the Taguchi method includes parameter levels’ design, orthogonal array, and factor and level effects. It offered a set of the scientific experimental procedures, which facilitates doctors and the medical staff to control important experimental factors and levels, and to achieve a reliable estimated value of factors in fewer experiments, the comparison of the full factorial design needs a huge of resources and time consumption (the full factorial design needs 81 sets of experimental combinations). The optimal factors and levels are established by a summary of the professional knowledge of doctors and the medical staff, which also provides an optimizing solution for intern doctor’ reference.

### Omega transformation

To validate the reproducibility of the optimal factor and level combination of A_1_B_3_C_2_D_2_ in order to facilitate the confirmation of the correctness of the experiment, we applied the omega transformation proposed by Dr. Taguchi and converted the ratio to a corresponding dB value, which was utilized to ensure quality in the product or manufacture. As the quality characteristics in this study were divided into good and defective (binary data), the omega transformation helped us to transform the binary data into dB to estimate the defect rate. The formula is as follows:

1 $$\varOmega \, = \,10 \cdot \log_{10} \left( {\frac{1 - P}{P}} \right)$$

The *P* in Eq. (1) is the defect rate of the selected optimal controlling factor and level. The conversion data values are as follows:

$$\begin{aligned} \bar{A}_{1} \, = \,23/90 = 0.26 \to \varOmega \, = \,4.54{\text{ dB}} \hfill \\ \bar{B}_{3} \, = \,24/90 = 0.27 \to \varOmega \, = \,4.32{\text{ dB}} \hfill \\ \bar{C}_{2} \, = \, 2 1/ 90 = 0. 2 3\to \varOmega \, = \, 5. 2 5 {\text{ dB}} \hfill \\ \bar{D}_{2} \, = \, 2 6/ 90 = 0. 2 9\to \varOmega \, = \, 3. 8 9 {\text{ dB}} \hfill \\ \bar{T}\, = \, 8 6/ 2 70 = 0. 3 2\to \varOmega \, = \, 3. 2 7 {\text{ dB}} \hfill \\ \end{aligned}$$

After these individual estimated values were calculated, we continued to calculate the estimated value of the optimal factor and level combination (A_1_B_3_C_2_D_2_), and the converted Ω values are as follows:

$$\begin{aligned} \varOmega ({\text{A}}_{ 1} {\text{B}}_{ 3} {\text{C}}_{ 2} {\text{D}}_{ 2} )\, - \,{ 3}\varOmega \mu &= 4.54 + 4.32 + 5.25+ 3.89 - 3*3.27 = 8.19\hfill \\ \end{aligned}$$

Therefore, the estimated value of the best condition is 8.19. Next, the estimated value was converted back to the original defect rate. When *Ω* = 8.19, the corresponding estimated defect rate is 13.17%. Later on, we conducted an experiment to further discuss whether the estimated value derived from the omega transformation matches the estimated defect rate after improvement.

2 $$P\, = \,\frac{1}{{1\, + \,10^{{\frac{\varOmega }{10}}} }}\, = \,13.17\%$$

### Prediction of the proposed combination of optimal factors and levels

#### Back-propagation network setting

This section discusses how the back-propagation network was utilized to predict whether the proposed combination of optimal factors and levels, A_1_B_3_C_2_D_2_, is stable with low margins of error. We used the Matlab 2012a software and inputted “nntool” in the command, after which the neural network parameter in this experiment was set, as shown in Tables 8 and 9.

**Table 8:** Neural network parameter setting

Setting items	Setting content
Network pattern	Back-propagation network
Input variables	4 units
Number of hidden layers	1 layer
Output variables	1 unit
Learning rate	0.1
Inertia item	0.1
Learning cycle	500
Learning rule	Delta rule

**Table 9:** Input and output variables setting

Input variables	Output variables
X1: Amount of anticoagulation at 500 units	Y1: Artificial kidney solidification data
X2: Velocity of blood flow at 300 ml/min	
X3: Dehydration volume at 2.5 kg	
X4: Autologous blood vessel as the vascular access type	

#### The convergence test

For reducing a human trial risk in the follow-up confirmation experiment (see “Confirmation experiment” section), we have to conduct a more rigorous test procedure. In this study, the back-propagation network, with high prediction accuracy and the fastest learning rate, was utilized thereby as the research tool. The back-propagation network needs a convergence test procedure for validating the experiment performance suggested by Hsu et al. [27], Du et al. [28], Mehra et al. [29], and Wang et al. [30].

**Fig. 7:**
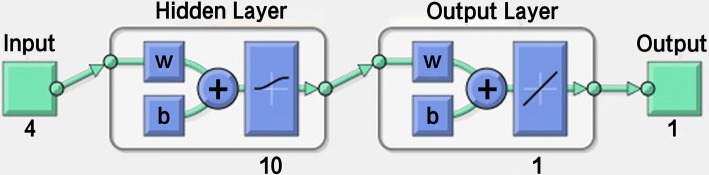
Training procedure computing simulation diagram

First of all, the Taguchi method uses four factors at three levels to conduct 270 experiments. Anticipating that the experimental data would be universal, correct, and average, and judging from the effect diagram of the average analysis, the purpose of the experiment was to reduce the error of the estimated value of artificial kidney solidification and to achieve convergence. This study used the Mean Squared Error (MSE) to analyze the training results. The definition of MSE is shown in formula (3):

3 $${\text{MSE}}\, = \,\frac{1}{Q}\sum\limits_{q = 1}^{Q} {(T_{q} \, - \,A_{q} )^{2} }$$

where $T_{q} \, - \,A_{q}$ (*T* as estimated value and *A* as actual value) represents the estimated value minus the actual value of the *q*th item to calculate the error value, and *Q* represents the number of estimated values. When the MSE approaches 0, the error value between the estimated value and the actual value becomes smaller, which means that the result of the prediction pattern is better. In this experiment, the data were obtained through the Taguchi method. Following the steps of the experiment, and through the optimal prediction procedures of the back-propagation network, the training procedure was developed, as shown in Fig. 7.

**Fig. 8:**
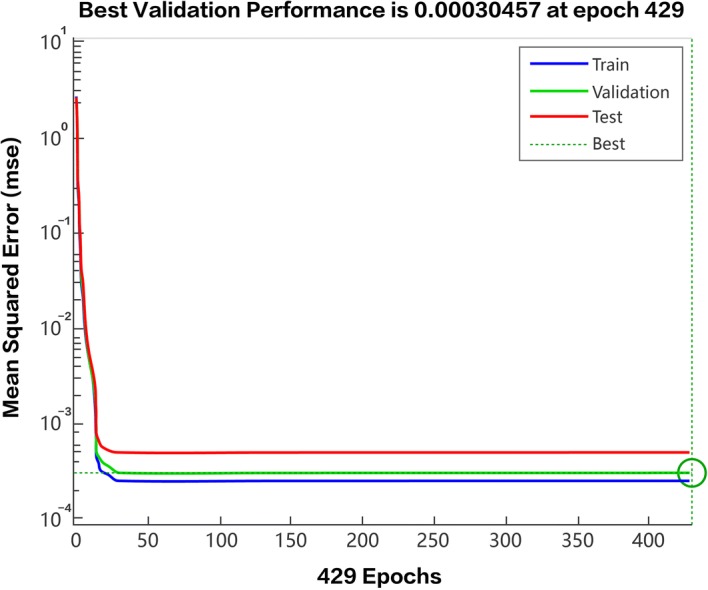
MSE cycle convergence diagram. The red line represents the testing performance; the green line represents the validation performance; the blue line represents the training sample performance

Secondly, the 270 sets of data that were analyzed in the experiment, which are in either input values or output values, were imported into the back-propagation network. The 270 data sets were divided for training 80% (216 samples), validation 10% (27 samples), and testing 10% (27 samples) by a setting of the back-propagation network. The setting of percentage of training, validation, and testing is suggested by Mehra et al. [29]. Through the operation procedure process of the back-propagation network, we obtained the following cycle convergence diagram in Fig. 8.

Thirdly, this study used MSE to judge whether the data had achieved convergence. The MSE cycle convergence diagram in Fig. 7 shows the error between the estimated value and the actual value, and when the target value of the validation performance (green line) is close to 0, it indicates that, as a result, the degree of convergence is better. Finally, in this study, the cycle convergence diagram (see Fig. 8), obtained using the back-propagation network, indicates that the error (MSE) of the training results of the back-propagation network is 0.00030457, and the number of network training iteration is 500. Both the testing sample and the training achieved the convergence effect at approximately the 37th iteration, and the testing performance, training performance, and validation performance all presented stable convergence. This conclusion indicates that, in this study, the back-propagation network, after carrying out repeated training, achieved consistent and stable results.

On the contrary, it has a deviation between the estimated value and the actual value in MSE cycle convergence diagram, which means that MSE cycle convergence is worse. It is suggested that the experiment should go back to the stage of small group discussion (please see the proposed model in Fig. 2).

### Confirmation experiment

This section discusses how this study conducted an actual hemodialysis procedure in the confirmation experiment, and illustrated the experimental results. The proposed combination of optimal factors and levels, A_1_B_3_C_2_D_2_, was utilized in the confirmation experiment following this condition. The doctors in the hospital were asked to carry out 270 hemodialysis procedures, and the number of artificial kidneys with a defective quality is 7. Therefore, the percentage of artificial kidneys with a defective quality is 12.9%, which is similar to the estimated defect rate of 13.17% (see the result of formula 2). This means that the optimal factor and level combination, derived from the Taguchi method, has good reproducibility in the hemodialysis experiment. It will be applied to the hemodialysis procedure of the patients after the confirmation experiment. The data are shown in Table 10.

**Table 10:** Reproducibility experimental data

Anticoagulation	Velocity of blood flow	Dehydration volume	Vascular access type	Detection value		
A_1_	B_3_	C_2_	D_2_	Defective quality	Good quality	Total
500 units	300 ml/min.	2.5 kg	Autologous blood vessel	35	235	270

### Optimization of the patient characteristics for physiological status

**Fig. 9:**
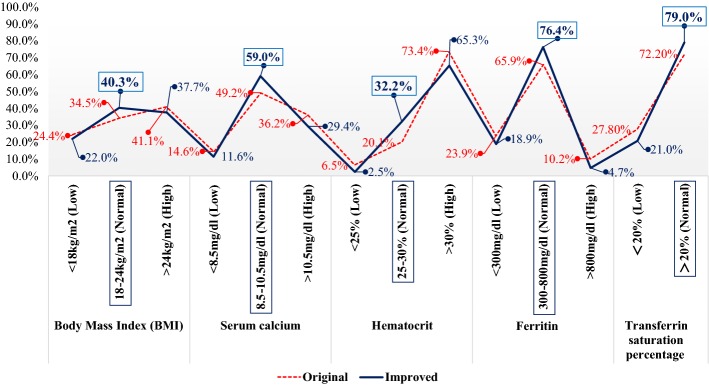
The optimization of patient physiological status

The combination of optimal factors and levels A_1_B_3_C_2_D_2_ is validated that can optimize the patients of kidney function. The subject 270 patients then experienced the experiment of the proposed optimal factor and level combination by this study at least half a year. We collected the data of the 270 patients’ characteristics for physiological status (see Fig. 9) optimized which are as follows: (1) BMI of the patient in normal rate (18–24 kg/m^2^) is improved from 34.5% (original) to 40.3% (improved); (2) serum calcium of the patient in normal rate (8.5–10.5 mg/dl) is improved from 49.2% (original) to 59.0% (improved); (3) hematocrit of the patient in normal rate (25–30%) is improved from 20.1% (original) to 32.2% (improved); (4) ferritin of the patient in normal rate (300–800 mg/dl) is improved from 65.9% (original) to 76.4% (improved); and (5) transferrin saturation percentage of the patient in normal rate (> 20%) is improved from 72.2% (original) to 79.0% (improved). The optimization of the five characteristics in physiological status might be partially from the positive influence of supplementation (e.g. vitamin B1 and vitamin D) and diet control to protect residual kidney function [31, 32].

## Conclusion

From our research, we learned that during the hemodialysis procedure carried out by medical staff, the solidification rate of the artificial kidney could result in detriment to the patients, such as loss of blood or dizziness. To enhance the survival rate of the patients undergoing hemodialysis, this study proposed an integrated model, which applied the Taguchi method, omega transformation, and back-propagation network to systematically identify the optimal parameters in terms of artificial kidney solidification and to improve the problem of artificial kidney solidification.

In practice, when the clinical practice in hospitals conducts quality improvement of hemodialysis, they often adopt quality control circle techniques; however, when the clinical practice encounters issues, such as having too many factors from which to choose and struggling with how to select the most important factors, they usually rely on the trial and error method to conduct experiments. In comparison with the current clinical practice, the combination of optimal factors and levels A_1_B_3_C_2_D_2_ has been confirmed by this study. We analyzed the results of previous studies and adopted the small group discussion method to systematically acquire the professional knowledge of doctors and the medical staff. Their opinions were collected and used to determine the important factors and levels affecting artificial kidney solidification and to validate the experiment variables. The study offered the doctors and nursing staffs with the optimizing experimental factors and levels, which allows them to minorly adjust the value of the levels (adjustable level: velocity of blood flow range as 200 ml/min to 300 ml/min, and dehydration volume range as 1.5 to 3.5 kg), the such method can quickly achieve ‘robust design’ for reducing the solidification rate of the artificial kidney, in accordance with patients’ physiological status. Meanwhile, the study also offers a prediction procedure by the back-propagation network, which helps doctors and nursing staffs to test the proposed combinations of optimal factors and levels are whether achieved consistent and stable results.

Next, this study took advantage of the Taguchi method to identify the significant factors and levels, leading to the identification of the optimal procedure parameter combination and predicted defect rate after improvement. The optimal factors and levels identified in this study are 500 units for the amount of anticoagulation, 300 ml/min for the velocity of blood flow, 2.5 kg for the dehydration volume, and autologous blood vessel for the artery vessel passage type. The validation experiment was carried out in hospital cases with 12.9% as the artificial kidney solidification rate. Artificial kidney solidification was indeed reduced by the proposed model. Meanwhile, the optimal factors and levels in this model improved the patient characteristics for physiological status in BMI, serum calcium, hematocrit, ferritin, and transferrin saturation percentage.

Secondly, this study discovered that many scholars had trained the experimental data, obtained through the Taguchi method, with the optimal prediction procedure of the BPN and received the optimal result that enhances the convergence speed and adaptability; however, after searching in the SDOS and IEEE for records of international journals within the last 5 years, we found no research articles that combined the count value as quality characteristics, as per the Taguchi method, with the BPN. Therefore, this study trained the experimental data, obtained based on the Taguchi method, on the optimal prediction procedure of the BPN, with 0.00030457 as the MSE. This can improve the convergence of the original solidification error and validate the influential key factors, verifying that the proposed model provides an effective analysis procedure.

By proposing an integrated model that successfully uses the Taguchi method, omega transformation, and back-propagation network, this study has made three important contributions: (i) it has greatly reduced the defect rate from 32% (original) to 12.9% (improved); (ii) during the experimental process, the parameters set by the Taguchi method were trained on the back-propagation network’s convergence pattern, and the number of training iterations was kept under 37 times with the convergence result as stable and linear, which means that the average errors of the Taguchi method and the BPN were verified to be quite small; (iii) the use of the Taguchi method and back-propagation network to identify the convergence effect and acquire a stable value of artificial kidney solidification to reduce errors in the estimated value in the prediction pattern was an optimal way, without wasting too much time and costs, to provide a reference for related business owners to make simple and convenient decisions.

The aims of this study were to reduce the solidification rate of artificial kidneys and conduct experiments on the cases. Before the improvement, the solidification rate of the artificial kidney was 32%; after the improvement, the solidification rate of the artificial kidney deceased to 12.9%. This matches the predicted defect rate of 13.17%, after omega conversion, and shows the correct rate of this study’s results. By summing up all of the aforementioned conclusions, we can confirm that our proposed model, integrating the Taguchi method, omega transformation, and back-propagation network to determine the optimal factor parameters for the hemodialysis procedure, indeed makes a great contribution to the field of medical care. Meanwhile, the proposed model can also be regarded as a standard operation procedure for quality improvement when the hospitals carry out clinical experiments. In other words, if hospitals apply the model proposed by this study, using the Taguchi method to control important experimental factors and levels and the back-propagation network to predict the correctness of the models, before they conduct any medical procedures, they may minimize the time and cost required for figuring out important factors and levels and thus optimize medical quality and stabilize treatments for optimizing patient health.

## Data Availability

The data are presented in the attachment. Please refer to Tables 4, 5, 6, 7, 8, and 9.

## Abbreviations

MSE: Mean Squared Error
BPN: back-propagation network
